# Current Situation of Heat Stress Studies on Kendo Players: A Scoping Review

**DOI:** 10.3390/jfmk9040219

**Published:** 2024-11-04

**Authors:** Hatsune Shishido, Masaharu Kagawa

**Affiliations:** 1 Institute of Nutrition Sciences, Kagawa Nutrition University, Saitama 350-0288, Japan; shl195@eiyo.ac.jp; 2 Sports Performance Research Institute New Zealand, Auckland University of Technology, Private Bag 92006, Auckland 1142, New Zealand; 3 School of Population Health, Curtin University, Perth, WA 6845, Australia; 4 School of Exercise and Nutrition Sciences, Queensland University of Technology, Brisbane, QLD 4001, Australia; 5 Faculty of Public Health, Mahidol University, Bangkok 10400, Thailand

**Keywords:** Kendo, heat stress, dehydration, global warming, Japanese, review

## Abstract

**Background/Objectives:** Global warming and the rise in the average temperature in recent years have increased the risk of heat stroke and also deteriorated performance among athletes. Kendo, a traditional Japanese martial art and also a competitive sport, is reported to have high incidents of heat stroke and related mortality. However, there is no heat management guideline for this specific sport to date and research on its heat management practices and risk factors for heat stroke are limited. The present study conducted a scoping review on studies focused on heat stress and the heat management practices of Kendo players. **Methods:** A literature search was conducted using five databases (PubMed, SCOPUS, Ichu-shi Web, CiNii, and Google Scholar) and also manually from the references of searched articles. **Results:** Overall, the studies that have investigated the heat stress of Kendo players are scarce and outdated. Of the 15 references that met screening criteria, 11 studies were conducted in a field setting. The vast majority were conducted on male university students and there was a lack of research on females and on different age groups. Common measures of heat management practices used in previous studies were weight changes before and after training (*n* = 14), body temperature (*n* = 9), and heart rate (*n* = 8). Only a few studies used multiple measures to determine heat stress. **Conclusions:** Considering the continuation of global warming and the increasing risk of heat stroke, further investigation on heat stress, its association with health and performance, and current heat management practices in Kendo players are warranted.

## 1. Introduction

Global warming and subsequent climate changes have been serious environmental and public health issues for decades, and Japan is no exception. The deviation of the average temperature during the summer has increased from 1.08 °C in 2010 to 1.76 °C in 2023 [1] and the annual temperature has been increasing at a rate of 1.25 °C/100 years since 1898 [2]. Furthermore, Japanese summers are characterized by high humidity due to the influence of the North Pacific High, which brings warm, moist air [3]. A study on regional differences in humidity’s effect on heat-related mortality has reported that muggy conditions tend to significantly impact the mortality risk in Japan [4].

Global warming and climate change influence, not only daily living, but also exercise and sports activities. Extreme weather often limits athletes’ training time and increases injury risk, with postponements and cancelations of competitions due to extreme heat also increasing [5]. A past study reported that, due to the rise in the average temperature, the number of cities capable of hosting the Summer Olympic Games will reduce to as low as 27% by the second half of the 21st century [6]. Exercising in a hot environment causes profuse sweating and increases the risk of dehydration, which affects the heat-dissipating function of the core body temperature and results in an excessive increase in body temperature. Inappropriate responses to dehydration may result in the decline of physical function and performance [7] and also increases the risk of exertional heat stroke [8]. The adequacy of fluid intake during training can be determined from the change in body mass measured before and after the training and it has been suggested that more than 2% of weight loss can impair the aerobic performance of athletes [9,10].

While exposure to extreme heat is well-known to deteriorate one’s health status and performance, a number of studies have reported that this can be prevented through adequate heat management before and during exercise [11,12,13,14]. Common heat management techniques include fluid intake, body cooling, and acclimatization [15]. It is also recommended to wear highly breathable and moisture-dissipating clothing with high heat dissipation and low heat retention [16]. Furthermore, the large exposure of body extremities is also considered important [16]. To date, national and international organizations such as the American College of Sports Medicine (ACSM), the National Athletic Trainers’ Association (NATA), and the International Olympic Committee (IOC) have proposed position stands that specifically focus on hydration [9,17,18]. In Japan, the Japan Sports Association (JSPO) published a guidebook for the prevention of heat stroke during sports activities [16]. In order to incorporate existing guidelines, some sports have modified their regulations to provide additional time for fluid intake and to make decisions for the postponement/cancelation of competitions [19,20,21,22,23,24,25] (Table 1). However, the feasibility of these recommendations is largely dependent on the characteristics of the participating sports. Sports that require their athletes to wear protective gear (e.g., American football and fencing) are reported to be highly vulnerable to deteriorate their performance and heat stroke as athletes experience impeded heat dissipation [26,27]. Past studies showed that athletes of different sports and competition levels are aware of the adverse effects of heat stress on their performance and health [11,28]. However, it has also been reported that many take inappropriate heat precautions, mainly due to training venues and timing, as well as due to a lack of knowledge of both the athletes themselves and the coaches [28].

Kendo (Japanese swordsmanship) is one of the traditional Japanese martial arts and a common sport in Japan, with more than 2.02 million competitors (with approximately 600 thousand female competitors), including junior high school and high school students [29]. In Kendo, players must wear traditional Japanese outfits (kendo-gi and hakama) that cover the vast majority of their surface area and a set of protectors (men [face protector], kote [gauntlets], do [breastplate], and tare [faulds]) that constitute a total weight of 4–6 kg [30]. On top of ordinary outfits and protectors, the All Japan Kendo Federation (AJKF) mandated the wearing of masks and also strongly recommended the use of mouth shield in 2020 to prevent the droplet transmission of Severe Acute Respiratory Syndrome Coronavirus 2 (SARS-CoV-2) [31]. Today, wearing masks is no longer mandatory but the AJFK still recommends either wearing masks or mouth shield [32]. As a result, Kendo players are experiencing high heat production during training and competitions; their heat dissipation is disturbed and they are also experiencing difficulty in fluid intake [33]. This puts Kendo players at a greater risk of developing heat stroke, and it has been reported that Kendo is one of the sports with high incidents of heat stroke and stroke-related death in junior high schools and high schools in Japan [34].

While Kendo has high incident and mortality risks of heat stroke, no heat management guidelines exist that are specific to this sport. In addition, compared to other “at risk” sports such as American football and fencing, studies that have explored current heat management practices and the factors that attribute to the development of heat stroke among Kendo players are limited. In the present study, the existing literature on heat stress and heat management practices among Kendo players were summarized to better clarify the current issues related to heat stress in this population.

## 2. Materials and Methods

**Figure 1:**
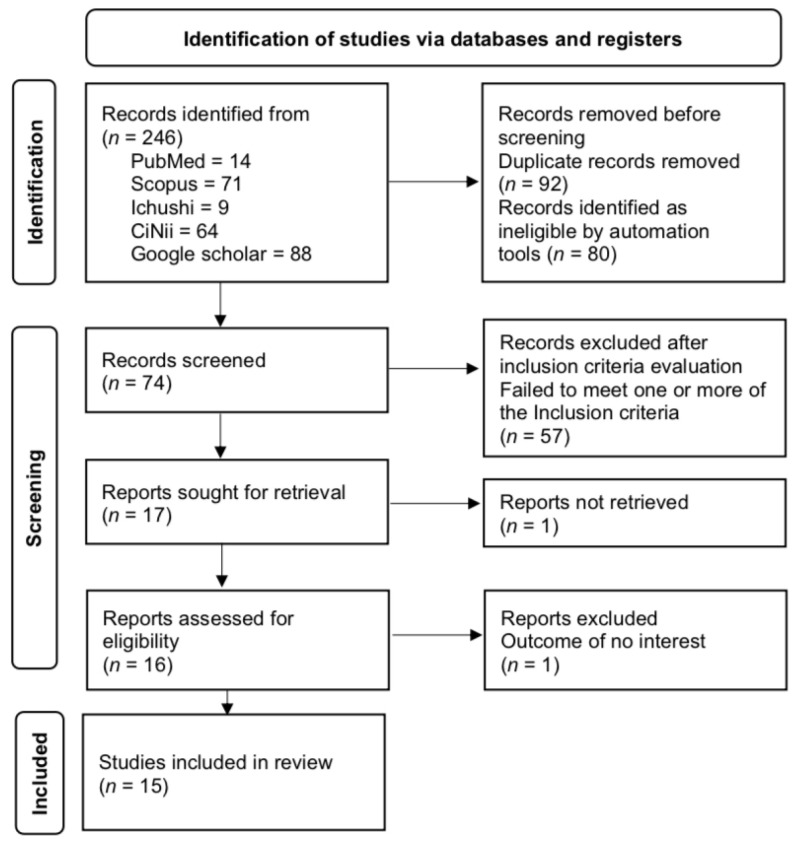
Selection process for the research articles (*n* = 15) included in this review.

The present study used a scoping review approach to systematically gather and map the existing research concepts and methodologies to identify gaps of knowledge [35]. With the research question, “What does the literature reveal about heat stress and heat management practices in Kendo players?”, a literature search was conducted using five database search engines (PubMed, SCOPUS, Ichu-shi Web, CiNii, and Google Scholar) and also manually from the references of the searched articles. This study followed the PRISMA extension for scoping reviews (PRISMA-ScR) guidelines to collect, select, and evaluate articles [35,36]. In this study, the inclusion of a broad range of literature was prioritized and, therefore, we did not conduct a bias risk check. The search for papers was conducted through 12 October 2024 with the following inclusion criteria: (1) articles published up to October 2024; (2) studies including healthy Kendo players; (3) studies on the risks of heat stroke (e.g., dehydration and increased body temperature); (4) accessible in full; and (5) published in Japanese or English. All the studies were included regardless of the sex, age, and ethnicity of those studied. Conference abstracts, articles with no full text counterpart, and articles with full text that was not available were excluded. The literature search was made using the keywords “Kendo” AND (“Heat stroke” OR “Heat illness” OR “Heat stress” OR “Heat exhaustion”) AND (“Thermoregulation” OR “Heat environment” OR “Hot environment” OR “Thermal stress”) AND (“Dehydration” OR “Hydration” OR “Water balance”). The searched articles were checked to remove any duplicates and then screened based on their titles and abstracts. Those found to meet the inclusion criteria were then checked thoroughly via reading the full text. After the removal of duplicates and screening, a total of 15 articles were found eligible to be included in the review (Figure 1).

## 3. Results

A total of 15 studies [33,37,38,39,40,41,42,43,44,45,46,47,48,49,50] remained after screening (Table 2). These studies were published between 1991 and 2024. Most of the studies (*n* = 12; 80.0%) were conducted on male university students and only one study [41] included females. However, this study combined results obtained from both genders and therefore it was unable to determine the presence of any gender differences in the results. There were no studies on young children and adolescents. None of the studies mentioned the inclusion of participants with a past history of heat stroke. In addition, apart from the two studies that clearly stated the acclimation of the participants [49,50], no other studies described the consideration of the heat acclimatization of the participants. Further, the procedure of acclimation in these two studies was only placing participants in the environment chamber for 30 min. Of the 15 studies, 9 studies were conducted on the field or replicated conditions during training [37,40,41,42,43,44,45,46,48] whereas four studies were conducted in a laboratory using facilities like an environmental chamber [33,39,49,50]. The remaining two studies reported experiments conducted in both field and laboratory settings [38,47]. The type and intensity of the exercise regimen varied between studies, ranging from bicycle ergometers (*n* = 4) to treadmills (*n* = 2) and different exercises that simulate actual training session. The total duration of the exercise load ranged from 4 to 120 min. Laboratory studies that used an environmental chamber also had different exercise periods of 4–60 min and a rest period of 5–30 min before entry/re-entry of the chamber.

Common measures for dehydration were body temperature (*n* = 9), heart rate (*n* = 8), weight changes (*n* = 14), blood (*n* = 7), and urine (*n* = 1) (Table 3). The most popular site to measure body temperature was tympanic or internal auditory canal temperature [39,42,44,47], followed by skin temperature [33,39], rectal temperature [43,47], external auditory canal temperature [49,50], and underarm temperature [42]. The body temperature measured at different sites provided different values. In addition, one study reported a rapid increase in the internal auditory canal temperature compared to the rectal temperature, suggesting a difference in the response to exercise depending on the site of the body temperature measurement [47]. All the studies conducted on the field (*n* = 5) reported an increase in body temperature after the training. A study conducted by Watanabe et al. [43] reported a continuous increase in the rectal temperature of all the participants during training, and a marked increase to 39 °C was observed during ji-geiko, a repetitive training aimed to master basic movements.

The literature search identified some studies that investigated factors’ impact on change in body temperature among Kendo players. Laboratory studies using a bike ergometer or a treadmill (*n* = 2) showed a significant increase in body temperature when exercise was conducted with Kendo wear and protectors compared to the session in light clothing (e.g., shorts, swimsuits) [33,39]. Satsumoto et al. [47] investigated the impact of materials of training wear (100% cotton and 95% polyester) on heat stress. From comprehensive assessments of skin and rectal temperatures, humidity within training wear, total perspiration rate, heart rate, and subjective comfortableness, polyester material was suggested to have lesser heat stress during Kendo training than cotton material. Further, a recent study that investigated the impact of a mask or face shield under a men [face protector] reported a significant increase in the external auditory canal temperature after exercise using a bicycle ergometer compared to the temperature measured while in seated a position without a mask or face shield (37.36 ± 0.22 °C vs. 37.23 ± 0.27 °C; *p* < 0.05) [49].

Some studies used heart rate as a measure of heat stress during Kendo training. However, past studies that investigated the impacts of training wear and protectors on heart rate showed inconsistent results [33,39]. In addition, no significant differences in heart rates were observed between different types of exercise (i.e., bike ergometer exercise and ji-geiko) of the same duration (60 min) [38], with the presence of fluid intakes during ji-geiko training [40], as well as with a mask or face shield under a men [face protector] [49].

A change in body mass before and after the training has been commonly used as an index of dehydration during exercise. The present study found 13 studies (15 experiments) that included a change in body mass (Table 4). Although the duration of exercise and the condition of fluid intakes varies, the range of dehydration as determined by a change in body mass was about 0.6–4.0%. Not all the studies clearly described the condition of fluid intake during the experiment. However, it was found that four experiments did not allow any fluid intake during the experiment [37,38,44] and six experiments allowed fluid intakes at restricted amounts and timings [40,41,46,47,49,50]. The type and temperature of fluid also varied between studies. While four experiments supplied water [40,42,46,48] and two experiments provided sports drinks [47,49,50], one experiment allowed participants to select either a sport drink or barley tea [41]. While not many provided details about the temperature of the supplied drinks, two experiments provided sports drinks at room temperature and four experiments provided cold drinks. One of the studies compared fluid intake via the use of a straw, which aided the participants in drinking without removing their face protector [42]. This study showed that the group using a straw had a higher fluid intake relative to perspiration compared to those drinking without straws (52.2% vs. 28.0%). However, both groups did not achieve the 80% relative to perspiration that is required to prevent heat illness and both the groups experienced significant body weight loss during training (over 2% in the straw-use group and over 3% in the comparison group), indicating their risk of heat illness and declined performance.

As an alternative measure of dehydration, serum volume, blood hematocrit, and vasopressin levels were used in five studies [37,38,39,40,46]. While the results of the blood hematocrit and vasopressin levels were inconsistent between the studies, three studies reported a reduction in blood serum volume after a training session [37,38,40]. In addition, Lue et al. investigated the oxidative state of serum albumin as an indicator of heat stress during a training camp. They reported a decrease in the average fraction of albumin in the reduced form of 13.9 ± 1.2% after the training camp and suggested the possible influences of physical exertion and high summer temperatures on oxidative stress in the participants [42]. Unlike the change in body mass and the analysis of blood samples, an analysis of urine samples such as urine specific gravity (USG) was only reported from one study [45]. The study reported a significant (*p* < 0.05) difference in USG measured before and after the training session [45]. In addition, from the observed relatively high USG from the pre-training assessment, the study also suggested possible chronic dehydration among Kendo players.

In the present literature search, a vast majority of studies evaluated heat stress and the risk of heat illness using a range of indices. In comparison, it was unable to identify many studies that utilized body cooling techniques to prevent heat illness. The only study found was a laboratory experiment that investigated the usefulness of palm cooling during training under heat conditions with their protectors [50]. This study reported that palm cooling significantly lowered the external auditory canal temperature measured at rest periods and also maintained power output during intensive exercise using a cycle ergometer compared to the control group. In addition, the present literature search did not find any studies that incorporated relationships between physiological parameters for dehydration and performance among Kendo players. Further, no studies were found that investigated the hydration status of Kendo players and their knowledge or attitudes toward heat stress.

## 4. Discussion

Kendo is a traditional Japanese martial art and is also recognized as a competitive sport. Due to its nature of its participants of wearing a uniform with a thick material and a set of protectors that covers almost the entire surface of their body, players are at a greater risk of dehydration and heat stress compared to other sports. However, the present study found only 15 studies that investigated heat stress among Kendo players. Of those, six studies were published in the 1990s. Considering the dramatic change in climate in recent years, it is likely that the climate conditions of these studies are not comparable to the climate today and, therefore, the results are outdated. In addition, the regulation of Kendo has been modified since the SARS-CoV-2 pandemic, including wearing a mask, face shield, and mouth shield; there is a need for more research that explores the impact of these new regulations on heat stress and associated health issues among Kendo players.

The present study revealed that the vast majority of the previous studies were conducted on male university students (*n* = 13; 86.7%) and studies on females and other age groups (i.e., children, adolescents and elderly) are completely lacking. Demographic information such as age and competition level are assumed to influence tolerance to heat stress. However, a limited number of studies reported the age, rank, and body composition of the participants. This may be because previous studies did not consider the importance of these variables in the management of heat stress, but the time constraints and subject burden of including a questionnaire and detailed body composition assessments may also be possible reasons. In either case, there is a gap of knowledge on the relationship between competition level and the risk of heat stress among Kendo players.

To date, a range of measures have been used to investigate heat stress and risks of heat stroke in Kendo players. However, previous studies vary in their protocols (e.g., the type, the duration and intensity of exercise, the site of body temperature measurement, and the condition of fluid intake) and therefore these results need to be interpreted with care. Among the measures used, a change in body mass before and after the training was found to be the most common measure for dehydration. Due to its convenience and its simple and non-invasive nature, this measure has been frequently used to assess the hydration status of athletes of other sports [16,51]. While this technique is popular, it is important to acknowledge that comparing body mass measurements before and after training sessions alone cannot detect the presence of dehydration prior to training. A number of studies reported the dehydrated status of athletes even before training [7,8,9]. Considering the possibility that Kendo players may also be dehydrated before their training [45], it may be recommended to examine the pre-training hydration status of Kendo players in future research.

In recent years, the inclusion of multiple measures for hydration status has been recommended [17,51,52]. In the present study, 6 out of 15 studies included multiple measures for hydration status. Although it is highly invasive and not always available for the field setting, all seven studies included a change in body mass and blood sample analysis. A possible reason for blood analysis being utilized in the studies on Kendo players may be due to its applicability as a measure for both hydration status and as heat stress via assessing blood volume and blood biomarkers. In addition, the difficulty in obtaining accurate measurements for Kendo may also be associated with the use of blood samples. Kendo is a dynamic and fast sport with physical contact that requires protectors to cover the entire surface. This makes it difficult to measure physiological indices while training or during the competition. Consequently, although it is invasive, blood sampling and its analyses may be useful to better understand the physiological status of Kendo players comprehensively.

The present study revealed that, unlike blood analyses, assessments on urine samples are less popular in studies on Kendo players. As far as a measure of hydration status, urine sampling is a more convenient, less invasive, and practical method in the field setting than blood sampling. The infrequent use of urine sampling in Kendo players may be partially explained by the popularity of the technique. The JSPO guidelines only introduce two methods (i.e., weight changes and self-reported thirst) for hydration assessments [16]. Urine samples can provide a number of useful indices for hydration status, via various attributes, including their volume, color, USG, and osmolality. Since indices from urine allow both objective (volume, USG, and osmolality) and subjective (color) assessments of the hydration status at the time of sample collection, this is more useful than the change in body mass, which cannot clarify hydration status prior to the training. The application of urine in the assessment of hydration, however, requires few considerations. While it has been considered ideal to use the first urination of the day [17], the most appropriate timing to best reflect the pre-training hydration status of athletes remains uncertain. A previous study on Kendo players used a urine sample collected just before the training instead of the first urination of the day [45]. In addition, urine color can be influenced by a number of factors including diet, health conditions like kidney diseases, prescribed medications as well as testing environment (e.g., lighting and color of walls), the material of the container, and also the perception of the examiner. Therefore, the validity of urine color should always be thought about and not used as the optimal marker of hydration status, especially for large-scale studies [53]. For the Japanese population, it may be suggested to use a Japanese-specific urine color chart, such as the one proposed by Kataoka et al. [54] or rate the sample using multiple charts. Nonetheless, an increase in the number of studies using indices from urine samples may provide additional knowledge on the usefulness of measures from urine samples. Therefore, further investigation on hydration status and heat stress among Kendo players using multiple measures including urine sample is warranted.

From the present literature search, only one study examining a cooling method to prevent heat stress during Kendo training under high-temperature conditions was found. Fujita and colleagues [50] reported the effectiveness of palm cooling in maintaining external auditory canal temperature and power output. In Kendo, cooling must be performed either during breaks when the protectors are removed, or with methods that can be applied while wearing the protectors. Although further investigation in a real training setting is necessary, their findings may suggest the potential of palm cooling as a convenient and practical technique to reduce heat stress and maintain performance among Kendo players during training. In other sports, the usefulness of a range of cooling techniques, including ice slurry ingestion, has been widely investigated [12,14,55]. While the present study could not identify any other studies that focused on the usefulness of cooling techniques in Kendo, further research on cooling techniques in Kendo players may be beneficial in the prevention of heat illness in this particular population.

It is important to note that the present study was unable to identify any studies that investigated the relationships between the risk of heat stress, including dehydration, of Kendo players and their performance. Although it has been suggested that a fluid loss of more than 2% of the body mass will result in the deterioration of performance in athletes in general [7,8], there is no study that confirmed this suggestion in Kendo. In addition, since this suggestion is based on the difference in body mass measured before and after training, there is a gap in knowledge regarding whether this general statement is applicable to Kendo players with consideration of their pre-training hydration status. Similarly, the present study did not find any studies that examined associations between knowledge on heat stress and the actual heat management practices among Kendo players. To date, studies on knowledge and attitudes on heat stress and heat management among athletes are still limited [28,56,57,58]. Considering that knowledge and attitudes on heat stress are important components of behavior, further investigation to understand the link between one’s knowledge of and actual behavior on heat management will be beneficial.

The present study was unable to find research that specifically addressed heat acclimation or acclimatization in Kendo. Further, the existing literature lacked information on participants with a history of heat stroke or prior exposure to heat acclimatization programs. It has been reported that effective heat acclimatization generally takes several days and must be carefully tailored to the specific demands of both the sport and the individual athletes [16,59]. In order to determine the benefits of appropriate heat acclimation or acclimatization on heat stress and the prevention of heat illness, further research on this topic is warranted. However, while some sports such as American football have heat acclimatization protocols that account for the sport’s unique challenges [11,59], no such acclimatization protocol exists for Kendo to date, even though the AJKF recommends a planned heat acclimatization of 1-10 days and no training during hot periods [60]. Therefore, for the safety and health of Kendo players as well as to support their performances, research that aims to develop and validate Kendo-specific heat acclimatization protocol may be required.

## 5. Limitations and Future Direction

While this scoping review provides a valuable overview of heat stress and its management in Kendo players, several limitations need to be acknowledged. In the present study, a literature search was conducted using only five databases that are readily available without cost. The inclusion of additional databases may alter the search results. In addition, the keywords used in the literature search might have caused us to overlook relevant studies. Specifically, although almost no study found in the present literature search clearly described the inclusion of “Heat acclimation” and “Heat acclimatization”, using these as keywords may enable researchers to find studies that focus on these topics. Further, the present review exclusively focused on studies solely conducted on Kendo players. Consequently, the present study was unable to incorporate findings observed from studies that included Kendo players as a part of the athletes from a diverse range of sports. Although the number of Kendo players may be small, if results were reported separately for athletes of different sports, the inclusion of these studies might have provided a more comprehensive picture. Acknowledging these limitations, the present study is the first study that applied a scoping review to better understand the current situation on heat stress and its management in Kendo. Findings from the present review may contribute to the advancement of research in this population.

From the present study, it was found that the majority of studies that investigated heat stress were outdated and primarily focused on young adult males. This suggests the need for further research that reflects the current training condition of Kendo players and to explore heat stress risks across diverse genders and age groups. In addition, wide variability in the protocol to assess dehydration and heat stress between studies hindered result comparisons. Moreover, the lack of studies that employed multiple measures to assess heat stress may have affected the reliability of the results. In order to provide valid and reliable outcomes, future research should include multiple measures of standard protocols. In particular, the present study revealed that the analysis of urine samples in Kendo players is completely lacking. Considering its advantage in that it enables the assessment of hydration status prior to training and the strong likelihood of Kendo players being dehydrated before training, the application of urine sample analysis may be recommended in future research for this population. Similarly, the present review did not identify studies that utilized bioelectrical impedance analysis (BIA). BIA is a simple, non-invasive technique that is readily available today to estimate body composition, including total body water. While the accurate estimation of total body water in Kendo players needs to be validated, as estimation is largely affected by the measurement condition and prediction equation installed in the BIA device, results reported by Ueda et al. [61] indicate its potential as an alternative technique to estimate hydration status. Furthermore, the present literature search found a limited number of studies that focused on the usefulness of cooling techniques as well as heat acclimation or heat acclimatization. Although this may be partly due to the keywords included for the literature search, there is a possibility that there is a gap of knowledge on these topics and therefore further research may be warranted. Similarly, studies that explored the relationships between dehydration, heat stress, and Kendo performance, including their cognitive functions, and the connection between the knowledge of heat stress management and behavioral outcomes were also not identified from the present study. Since the absence of performance metrics limits the applicability of these findings, it is recommended to conduct holistic research that investigates relationships, not only regarding physical conditions and cognitive functions, but also the knowledge of dehydration and heat stress on the improvement of Kendo performance.

## 6. Conclusions

Kendo is a martial art and sport with a high risk of heat stress. This was the first study that searched and reviewed existing studies that investigated the risk of heat stress and its management of Kendo players. The results showed that studies on Kendo players were limited in number, and many were outdated. In addition, techniques used in previous studies were limited but varied in protocols and insufficient descriptions. Considering the number of limitations of existing studies and the increasing priority of heat management practice due to global warming, further investigations on heat stress, its association with health and performance, and current heat management practices on this population are warranted.

## Figures and Tables

**Table 1:** Examples of sports in Japan that modify competition rules to protect against heat stress.

Sports	Limitations Due to Ambient Temperature	Extended/Additional Breaks	Water/Cooling Breaks
Lacross [19]	Postpone on principle if the WBGT is 31 °C or higher If a decision needs to be made the day before, use a predicted WBGT of 33 °C or higher by 17:00 the previous day	QB: extended to 7′ from July–September (usually 2′) Halftime: can be extended to 15′ in certain games	In principle, not implemented May be implemented once per match in the event of adverse conditions
American football [20,21]	Avoid practice/games: DBT > 30 °C from 12:00 to 15:00 between 7/20 and 8/20 Cancel games: WBGT > 31 °C or NDB > 35 °C Consider heat stroke prevention: WBGT > 27 °C or DBT> 30 °C	NR	Every 6′ WB, or more if necessary
Soccer [22]	Cancel or postpone on principle if the WBGT is 31 °C or higher Conduct CB or WB if the WBGT is 28 °C or higher Conduct WB for Type 3 matches if the WBGT is 25 °C or higher Conduct CB for Type 4 matches if the WBGT is 25 °C or higher	NR	30″ to 1′ WB 3′ CB: 2times (once in the first half and once in the second half)
Softball/baseball [23]	Cancel or postpone on principle if the WBGT is 31 °C or higher Introduce a cooling break if a WBGT of 31 °C or higher is expected during the match	NR	Conduct 5′ WB if the WBGT is 28 °C or higher, up to 2 times 5′ WB may also be conducted if the defensive time for each inning exceeds 20 min Conduct 5′ WB if the WBGT is 25 °C or higher, provided the defending time exceeds 20′
Rugby [24]	For children of elementary school age and younger Severe warning if the WBGT is 28 °C or higher Discontinue exercise on principle if the WBGT is 31 °C or higher	For 7-man teams, consider extending halftime	3′ WB: 2 times (once in the first half and once in the second half)
Tennis [25]	Recommend replenishing water and salt during end changes if the WBGT is 25 °C or higher Recommend frequent rest, fluid and salt supplementation, and provide means for body surface cooling if the WBGT is 28 °C or higher Apply heat rules and modify play if the WBGT is 30.1 °C or higher When the WBGT reaches 30.1 °C, begin considering a temporary suspension of play within 90 min Suspend play on principle if the WBGT is 32.2 °C or higher	WBGT ≥ 30.1 °C: if requested by either player, 10′ break is allowed between the 2 and 3 set in 3 set matches	NR

″, second; ′, minute; NR, not recorded; WB, water breaks; CB, cooling breaks; QB, quarter breaks; NDB, natural dry bulb temperature.

**Table 2:** Summary of accepted papers.

Author [Citation Number]	Location	Year	Study Design	N (Gender)	Participants	Age (Years)	Participants Characteristics
Waku et al. [37]	Japan	1991	Field	5(M)	University students	20–22	Height: 167.5–177.2 cm, Body mass: 71.2–76.2 kg, BMI: 22.7–26.5 kg/m^2^
Waku et al. [38]	Japan	1992	Field/Laboratory	10(M)	University students	19–23	Height: 167.5–177.2 cm, Body mass: 63.3–79.1 kg BMI: 19.9–27.7 kg/m^2^
Waku et al. [39]	Japan	1994	Laboratory	4(M)	University students	20–21	Height: 170.3–178.5 cm, Body mass: 71.5–79.0 kg BMI: 23.3–24.8 kg/m^2^
Waku et al. [40]	Japan	1995	Field	5(M)	University students	21–23	Height: 167.0–178.0 cm, Body mass: 63.3–77.6 kg BMI: 20.0–27.8 kg/m^2^
Saito et al. [33]	Japan	1998	Laboratory	5(M)	University students	20.0 ± 1.0	Height: 176.2 ± 4.1 cm, Body mass: 70.8 ± 3.3 kg
Kozawa et al. [41]	Japan	1999	Field	22 (M15/F7)	University students	NR	Freshman, Height: 177.2 ± 5.0 cm, Body mass: 61.6 ± 10.3 kg Sophomore, Height: 169.1 ± 10.4 cm, Body mass: 68.1 ± 13.9 kg Junior, Height: 177.6 ± 3.0 cm, Body mass: 76.3 ± 4.9 kg Senior, Height: 171.4 ± 5.9 cm, Body mass: 68.4 ± 5.0 kg
Lue et al. [42]	Japan	2009	Field	10(M)	University students	(1) 19.8 ± 0.7 (2) 20.0 ± 0.5	(1) 171.0 ± 1.3 cm, Body mass: 64.7 ± 1.4 kg (2) 170.6 ± 1.5 cm, Body mass: 64.2 ± 3.1 kg
Watanabe et al. [43]	Japan	2011	Field	3(M)	University students	22.1 ± 0.32	Height: 175.6 ± 4.59 cm, Body mass: 68.8 ± 1.4 kg BMI: 21.5–23.0 kg/m^2^
Rossi et al. [44]	Brazil	2011	Field	12(M)	Practitioners from an institute of martial arts in São Paulo	26.0 ± 6.2	Height: 1.8 ± 0.03 m, Body mass: 78 ± 13.7 kg BMI: 24.12 ± 4.03 kg/m^2^, Body fat: 15.7 ± 5.1%
Yasuda et al. [45]	Japan	2012	Field	21(M)	University students	19.7 ± 1.1	Height: 170.2 ± 6.1 cm, Body mass: 68.8 ± 10.4 kg BMI: 23.8 ± 3.4 kg/m^2,^ Body fat: 16.7 ± 11.8%
Kubo et al. [46]	Japan	2012	Field	7(M)	Healthy adults	23.4 ± 0.8	Height: 175.9 ± 4.5 cm, Body mass: 66.0 ± 5.7 kg
Satsumoto et al. [47]	Japan	2013	Field/Laboratory	6(NR)	University students	20.7 ± 1.2	Height: 173.2 ± 4.8 cm, Body mass: 66.5 ± 4.6 kg
Yoshida et al. [48]	Japan	2023	Field	9(M)	University students	20.4 ± 1.3	Body mass: 66.5 ± 9.7 kg, BMI: 22.6 ± 2.3 kg/m^2^
Fujita et al. [49]	Japan	2023	Laboratory	8(M)	University students	19.1 ± 0.8	Height: 172.6 ± 6.2 cm, Body mass: 74.2 ± 8.9 kg
Fujita et al. [50]	Japan	2024	Laboratory	8(M)	University students	19.1 ± 0.8	Height: 172.6 ± 6.2 cm, Body mass: 74.2 ± 8.9 kg

M, males; F, females; NR, not recorded; –, range; mean ± SD.

**Table 3:** Key results of accepted papers.

Author [Citation Number]	Study Design	Climate	Exercise	Duration	Conditions Comparison	Parameters	Main Outcomes
Waku et al. [37]	Field	NDB: 30.4 °C (29.0–31.7 °C)	60′ ji-geiko (75%MHR)	30′ rest (sitting) 60′ ji-geiko (10′ × 6) 30′ rest (sitting)	(1) Lower body negative pressure test Designed to reduce the left ventricular end–diastolic dimension to the same degree as Kendo practice	Body mass, HT, BV, HR, BP, hemodynamics	Body mass: △↓, HT and BV: △↑ Left ventricular end-diastolic diameter and left atrial diameter: △↓ Stroke volume, ejection fraction, and shortening fraction: △↓ Ratio of left ventricular systolic end-Wall stress to left ventricular systolic end-volume index: △↑ Similar results were obtained for (1)
Waku et al. [38]	Field	NDB: 30.2 ± 1.0 °C NWB: 24.9 ± 2.1 °C RH: 70.2 ± 9.1% WBGT: 26.5 ± 1.9 °C	(1) 60′ ji-geiko (75%MHR)	30′ rest (sitting) 60′ Ji-geiko (10′ × 6) 30′ rest (sitting)	Comparison of (1) and (2) heat risks	Body mass, HT, HR SPC	Body mass: △↓after both exercises, with △↓ in (1) than (2) HT and TP: △↑ after both exercises, with △↑ in (1) than (2) HR: △↑ after both exercises, but △→ between (1) and (2)
	Laboratory	NDB: 36.5 ± 0.5 °C NWB: 27.0 ± 1.7 °C RH: 48.6 ± 7.3% WBGT: 30.7 ± 3.3 °C	(2) 60′ bicycle ergometer (70%MHR) Wearing shorts only over swimming trunks	30′ rest (supine position) 60′ exercise 30′ rest (supine position)			
Waku et al. [39]	Laboratory	(1) NDB: 25.4 ± 0.5 °C NWB: 21.2 ± 1.8 °C (2) NDB: 30.5 ± 0.4 °C NWB: 23.6 ± 1.3 °C (3) NDB: 30.5 ± 0.5 °C NWB: 24.2 ± 1.4 °C	60′ bicycle ergometer (135 ± 23-Watt, 90%VT) (62%MHR)	30′ rest (sitting) 60′ exercise 30′ rest (sitting)	(1) Wearing only shorts at room temperature (2) Wearing only shorts in hot environment (3) Wearing kendo-gi and protectors in a hot environment	Body mass, Ty, Tsk, HR, BP, HT, CA, SPC, Posm, ADH	Tre, Tsk: during exercise △↑ in trial (3) than in (1) and (2) △→ between the three trials: Body mass, HR, BP, HT, SPC, Posm, CA, ADH
Waku et al. [40]	Field	Day1 NDB: 30.7 ± 0.5 °C NWB: 24.8 ± 1.4 °C RH: 61 ± 8% WBGT: 26.5 ± 1.1 °C Day2 NDB: 29.3 ± 0.1 °C WB: 26.3 ± 0.1 °C RH: 79 ± 1% WBGT: 27.2 ± 0.1 °C	60′ ji-geiko	30′ rest (sitting) 60′ ji-geiko (10′ × 6) 30′ rest (sitting)	Water intake (1) or not (2)	Body mass, HT, BP, SPC, ADH, HR, hemodynamics, myocardial contractility	Body mass (with or without water intake): △↓ HT and SPC: △↑ ((1) and (2) △→) (2)Left atrial diameter, left ventricular end-diastolic diameter, stroke volume, left ventricular ejection fraction, left ventricular internal diameter shortening rate △↓ than (1) Ratio of left ventricular end systolic volume index to left ventricular end systolic wall stress is (2) after practice △↑, (1) △→
Saito et al. [34]	Laboratory	(1) NDB: 30.3 ± 0.2 °C NWB: 24.9 ± 0.2 °C RH: 64.8 ± 1.4% WBGT: 26.5 ± 0.2 °C (2) NDB: 30.0 ± 0.2 °C NWB: 25.5 ± 0.5 °C RH: 69.8 ± 2.7% WBGT: 26.9 ± 0.4 °C	15′ treadmill exercise (9.5 ± 0.8 km/h, 90%VT)	30′ rest (sitting) 15′ exercise 5′ rest (sitting)	(1) Wearing only short pants (2) Wearing kendo-gi and protectors	Body mass, Tre, Tsk, HT, ADH, Posm, HR, BP, VO_2_	Body mass: (1) and (2) △↓, WC △↓ in (2) than in (1) Ty and Tsk: △→ in (1), Ty and Tsk△↑ in (2) HR and oxygen consumption: during exercise △↑ in (2) than in (1)
Kozawa et al. [41]	Field	NDB: 31.4 ± 1.5 °C RH: 70.9 ± 4.3% WBGT: 28.1 ± 1.2 °C	143.1 ± 93.4′ Summer training camp (1) 9:00–11:30 kihon-geiko, kakari-geiko, gokaku-geiko (2) 14:30–17:30 Shiai-geiko, gokaku-geiko, kakari-geiko	30.6 ± 32.9′ kihon-geiko 56.9 ± 11.3′ gokaku-geiko 10.0 ± 0′ kakari-geiko 45.6 ± 49.2′ match 76.9 ± 17.1′ rest Interval	Comparison by Grade	Body mass, WI	WI: 1477.1 ± 685.7 mL (Before: 201.7 ± 256.1 mL, during: 320.3 ± 178.9 mL/h After: 537.0 ± 347.5 mL) Real body weight loss corrected by WI: 2.4 ± 1.1 kg Body mass loss after training: 0.9 ± 0.8 kg Fluid retention rate: 40.1% Significant regression between WI and real body weight loss (*p* < 0.05)
Lue et al. [42]	Field	WBGT: Morning/Afternoon Day 1: -/ 27.11 ± 0.1 °C Day2: 25.1 ± 0.8 °C/ 26.7 ± 0.3 °C Day3: 22.5 ± 0.7 °C/ 25.5 ± 0.7 °C Day4: 25.8 ± 0.4 °C/ 26.2 ± 0.2 °C	Summer training camp Morning (about 150′) Afternoon (about 180′) Only the afternoon practice was held on the first day, 7 sessions over 4 days	Amount of practice per day: 50′ kihon-geiko 110′ ji-geiko 20′ kakari-geiko 120′ shiai-geiko	Water high-intake group (1) or standard-intake group (2)	Body mass, WI, Ty, Tax, HSA	Body mass decrease (%): (1) 2.0 ± 0.4%, (2) 3.0 ± 0.2% ((1) △↓than (2)) WI: (1) 1.3 ± 0.2 kg, (2) 0.7 ± 0.1 kg ((1) △↑than (2)) Ty and Tax: No significant difference in temperature changes between (1) and (2). Additional water did not significantly suppress body temperature increases, and while it helped maintain body fluid levels, it was insufficient to prevent heat stroke.
Watanabe et al. [43]	Field	Day 1: NDB: 29.3 ± 0.875 °C RH: 71.6 ± 3.34% WBGT: 27.7 ± 0.65 °C Day 2: NDB: 32.1 ± 1.16 °C RH: 65.6 ± 4.93% WBGT: 29.5 ± 0.64 °C Day 3: NDB: 28.6 ± 0.34% RH: 56.0 ± 6.08% WBGT: 28.6 ± 0.34 °C	85′ Kendo practice (91.4%VO_2_max)	Day1–2 15′ warming-up, suburi 30′ kihon-geiko 40′ Ji-geiko Day3 15′ warming-up, suburi 30′ kihon-geiko 5′ kakari-geiko 5′ rest 40′ ji-geiko		Tre, HR	HR: Range of variability was consistent across all measurements. Tre: Rapid increase correlated with a steady rise in the trough level of rapidly fluctuating HR over a short period. All measurements with above 39 °C demonstrated sustained hyperthermia following exercise.
Rossi et al. [44]	Field	NDB: 24.1 ± 2.5 °C RH: 73 ± 8.5%	120′ Kendo practice	10′ warming-up 30′ kihon-geiko (40% VO_2_max) 20′ kakari-geiko (70% VO_2_max) 40′ gokaku-geiko (55% VO_2_max) 20′ shiai-geiko (70% VO_2_max)		Body mass, Ty	Body mass: △↓Ty: △↑in post than pre-exercise (*p* < 0.01) Sweat rate: 0.35 L·h^−1^ (95% CI = [0.299; 0.400]) Water loss: 0.946% (95% CI = [0.694; 1.174])
Yasuda et al. [45]	Field	WBGT: 19.4–21.9 °C	120′ Kendo practice	5′ warming-up 10′ suburi 15′ kirikaeshi 40′ kihon-geiko 25′ ji-geiko 20′ kakari-geiko 5′ kirikaeshi		USG, Ths, RPE	USG: △↑ after than before exercise, but also high at pre-exercise Ths and RPE: significant main effects for time were found (*p* < 0.05), but there were no significant main effects for phase or interactions
Kubo et al. [46]	Field	NDB:31,0 ± 1.9 °C RH: 60.0 ± 5.2% WBGT: 27.0 ± 1.3 °C	80′ menu based on normal practice content (70%MHR)	10′ suburi 40′ kirikaeshi, kihon-geiko, uchikomi 30′ gokaku-geiko	Crossover test between ingested (1) and non-ingested water (2)	Body mass, Blood fluidity, platelet aggregation	Blood fluidity in both groups △↓, following kendo practice. 30′ after kendo practice, both groups had recovered (the level of recovery △↑ in (1) than in (2))
Satsumoto et al. [47]	Field	NDB: 30.3 ± 1.0 °C WBGT: 27.3 ± 0.6 °C	25′ shortened menu like normal practice	5′ rest 10′ warm-up, suburi 15′ kirikaeshi, free practice, kirikaeshi 15′ rest	Wearing 2 types (COT, PET) of kendo-gi 20′ treadmill exercise	Ty, Tsk, Tre, HWC, Total perspiration rate, sympathetic nerve index	Tsk of COT △↑ than in PET (field test)
	Laboratory	NDB: 30 °C RH: 65%	20′ treadmill exercise	5 ‘rest 5 ‘walk 5 ‘fast walking 10 ‘running 20 ‘rest	Wearing 2 types of kendo-gi 25′ shortened menu like normal practice	Body mass, Tsk, Ty, Tre, HWC, Total perspiration rate, sympathetic nerve index	Tsk, Tre, Ty, total sweating rate, HWC of COT △↑ than in PET (chamber test)
Yoshida et al. [48]	Field	Summer NDB: 29.6 ± 0.8 °C RH: 73.3 ± 2.1%	135 ± 19′ Kendo Practice (Warming-up, three-step drill, wazageiko, rest, ji-geiko)	NR	Seasonal comparison (Spring, Winter) The parameters associated with water balance after adjusting for energy expenditure were assessed	Body mass, WI	Amount of sweating and WI, and WI rate: summer △↑ than other seasons Rehydration rate: 28% in spring, 39% in summer, and 22% in winter
Fujita et al. [49]	Laboratory	NDB: 32.9 ± 0.5 °C RH: 46.8 ± 2.0% WBGT: 28.1 ± 0.2 °C	4′ bicycle ergometer × 2 (7.5% body weight load) Wearing kendo-gi and protectors	30′ rest (sitting) in heat for heat acclimation 20″ full speed pedaling, 10″ rest × 8 15′ rest (sitting)	(1) Wearing a mask and face shield (2) Without mask and face shield	Exerted power, Te, HR, RPE	In the conditions of (1), Te: △↑during the rest period between exercises than in (2) Exerted power: △↓ during exercise after rest period than in (2) RPE and HR: △→
Fujita et al. [50]	Laboratory	WBGT: (1) 28.1 ± 0.2 °C (2) 28.2 ± 0.1 °C	4′ bicycle ergometer × 2 (7.5% body weight load) Wearing kendo-gi and protectors	30′ rest (sitting) in heat for heat acclimation 20″ full speed pedaling, 10″ rest × 8 15′ rest (sitting) or 10′palmar cooling and 5′ rest 20″ full speed pedaling, 10″ rest × 8	Icing during breaks or not (1) 15′ rest (sitting) (2) 10′palmar cooling and 5′ rest Masks and face shields were worn under both conditions	Exerted power, Te, HR, RPE	An interaction was observed between the two conditions regarding Te changes during rest, with the (2) condition significantly lower than the (1) condition after 6′rest In the (2), the decrease in power during all-out pedaling was significantly smaller than in the (1) condition

–, range; ±, SD; ′, minute; ″, second; NWB, natural wet bulb temperature; NDB, natural dry bulb temperature; WBGT, wet bulb globe temperature; RH, relative humidity; Tsk, skin temperature; Tre, rectal temperature; Ty, tympanic temperature; Tax, axillary (underarm) temperature; HT, hematocrit; HR, heart rate; BP, blood pressure; ADH, antidiuretic hormone; VT, ventilatory threshold; VO_2_, oxygen consumption; Posm, serum osmolarity; WI, water intake; HSA, human serum albumin; △, significantly; ↑, higher; →, indifferent; ↓, lower; ji-geiko, a training method that involves practicing techniques freely in a simulated match; suburi, practice swinging shinai; kirikaeshi, striking the left and right men target points in succession practicing centering; kihon-geiko, basic movements; kakari-geiko, short intense attack practice which teaches continuous alertness and readiness to attack, as well as building spirit and stamina; gokaku-geiko, a training method that involves practicing with a partner of roughly equal skill level.

**Table 4:** Study protocol for hydration.

Author [Citation Number]	Study Design	Exercise/Conditions	Restrictions on Fluid Intake	Amount of Sweat(g)/Sweat Rate (%)
Waku et al. [37]	Field	60′ ji-geiko	No fluid intake	At the end of exercise: 3.7% At the end of 30′: 4.0%
Waku et al. [38]	Field	60′ ji-geiko	No fluid intake	At the end of 30′: 2.3 ± 0.2%, (△↑ than bicycle ergometer)
	Laboratory	60′ bicycle ergometer	No fluid intake	At the end of 30′: 1.4 ± 0.1%
Waku et al. [39]	Laboratory	60′ bicycle ergometer	No clear statement	At the end of 60′: (1) Wear only short pants at room temperature 0.8 ± 0.3% (2) Wear short pants only in hot environment 0.8 ± 0.3% (3) Wear kendo-gi and armor in hot environment 1.3 ± 0.3%
Waku et al. [40]	Field	60′ ji-geiko	Cold water at 9 °C 500 mL (30′ before starting) 200 mL (15′ after starting the protocol)	(1) Conducted with water intake (2) Conducted without water intake At the end of exercise: (1) 1.4 ± 0.3 kg, (2) 0.8 ± 0.3 kg At the end of 30′: (1) 1.7 ± 0.4 kg, (2) 1.2 ± 0.4 kg At the end of 60′: (1) 1.8 ± 0.4 kg, (2) 1.4 ± 0.4 kg
Saito et al. [33]	Laboratory	15′ treadmill exercise	No clear statement	At the end of 60′: (1) Wearing only short pants 1.2 ± 0.2% (2) Wearing kendo-gi and armor 2.6 ± 9.4%, (△↑ than (1))
Kozawa et al. [41]	Field	Kendo practice (143.1 ± 93.4′)	Cold drinks (sports drink or barley tea) Beverages were freely available before, during, and after the practice	Real body weight loss, corrected for fluid intake 2.4 ± 1.1 kg, Weight loss after training 0.9 ± 0.8 kg
Lue et al. [42]	Field	150–180′ Kendo practice	Tap water at 7 °C (1) Additional drinking opportunities during short breaks (3 times in the morning, 3–5′ in the afternoon) (2) Limited to set rest periods (once in the morning, twice in the afternoon)	(1) 2.8 ± 0.1 kg, (2) 2.7 ± 0.1 kg (△=)
Rossi et al. [44]	Field	120′ Kendo practice	No fluid intake	0.946% (95% CI = [0.694; 1.174])
Kubo et al. [46]	Field	80′ Kendo practice	Mineral water at 10 °C 500 mL (after uchikomi-geiko) No food or fluids for 2 h prior to the experiment	At the end of exercise: Non water intake 1285.7 ± 203.5 g, Water intake 564.3 ± 419.3 g
Satsumoto et al. [47]	Field	25′ Kendo practice	No clear statement	No clear statement
	Laboratory	20′ treadmill exercise	200 mL sports drink Just before the experiment began	
Yoshida et al. [48]	Field	135 ± 19′ Kendo practice	Tap water Beverages were provided to participants before the practice, and they were free to drink as much as they wanted throughout the session	At the end of exercise: 1805 ± 437 g, 2.70 ± 0.48%
Fujita et al. [49]	Laboratory	4′ bicycle ergometer	Sports drink (room temperature) 500 mL (120′ before the experiment) 150 mL (Before entering the laboratory)	At the end of exercise: 0.48 ± 0.18 kg, 0.63 ± 0.18%
Fujita et al. [50]	Laboratory	4′ bicycle ergometer	Sports drink (room temperature) 500 mL (120′ before the experiment) 150 mL (Before entering the laboratory)	At the end of exercise: (1) 0.63 ± 0.18%, (2) 0.49 ± 0.17% (△=)

′, minute; –, range; ±, SD; NR, not recorded; ji-geiko, a training method that involves practicing techniques freely in a simulated match; uchikomi-geiko, a training method involving repeatedly striking a partner, aiming for designated target areas, while emphasizing the correct form and technique of basic strikes; △, significantly; ↑, higher; →, indifferent; ↓, lower.
